# Human transcriptional signature of protection after *Plasmodium falciparum* immunization and infectious challenge *via* mosquito bites

**DOI:** 10.1080/21645515.2023.2282693

**Published:** 2023-11-27

**Authors:** Marie Mura, Burook Misganaw, Aarti Gautam, Tanisha Robinson, Sidhartha Chaudhury, Neha Bansal, Andrew J. Martins, John Tsang, Rasha Hammamieh, Elke Bergmann-Leitner

**Affiliations:** a Immunology Core, Biologics Research & Development, WRAIR-Walter Reed Army Institute of Research, Silver Spring, MD, USA; b Host-Pathogen Interactions, Microbiology and Infectious Diseases, IRBA-Institut de Recherche Biomédicale des Armées, Brétigny-sur-Orge, France; c Medical Readiness Systems Biology, Center for Military Psychiatry and Neuroscience, Walter Reed Army Institute of Research, Silver Spring, MD, USA; d Vysnova Inc, Landover, MD, USA; e Center of Enabling Capabilties, WRAIR-Walter Reed Army Institute of Research, Silver Spring, MD, USA; f Multiscale Systems Biology Section, Laboratory of Immune System Biology, NIAID, NIH, Bethesda, MD, USA; g NIH Center for Human Immunology, NIAID, NIH, Bethesda, MD, USA

**Keywords:** Malaria, vaccine, human controlled malaria infection, protection, transcriptomic

## Abstract

The identification of immune correlates of protection against infectious pathogens will accelerate the design and optimization of recombinant and subunit vaccines. Systematic analyses such as immunoprofiling including serological, cellular, and molecular assessments supported by computational tools are key to not only identify correlates of protection but also biomarkers of disease susceptibility. The current study expands our previous cellular and serological profiling of vaccine-induced responses to a whole parasite malaria vaccine. The irradiated sporozoite model was chosen as it is considered the most effective vaccine against malaria. In contrast to whole blood transcriptomics analysis, we stimulated peripheral blood mononuclear cells (PBMC) with sporozoites and enriched for antigen-specific cells prior to conducting transcriptomics analysis. By focusing on transcriptional events triggered by antigen-specific stimulation, we were able to uncover quantitative and qualitative differences between protected and non-protected individuals to controlled human malaria infections and identified differentially expressed genes associated with sporozoite-specific responses. Further analyses including pathway and gene set enrichment analysis revealed that vaccination with irradiated sporozoites induced a transcriptomic profile associated with Th1-responses, Interferon-signaling, antigen-presentation, and inflammation. Analyzing longitudinal time points not only post-vaccination but also post-controlled human malaria infection further revealed that the transcriptomic profile of protected vs non-protected individuals was not static but continued to diverge over time. The results lay the foundation for comparing protective immune signatures induced by various vaccine platforms to uncover immune correlates of protection that are common across platforms.

## Introduction

Despite decades of intensive research, a highly effective vaccine conferring sterile protection to malaria is not yet available. Still, lots of progress have been made recently and the RTS,S/AS01B (Mosquirix®) journey,^1^ started in 1984, led to the historic recommendation of a widespread use for children in regions with moderate to high *Plasmodium falciparum* (*Pf*) malaria transmission by the World Health Organization in 2021 (Malaria vaccine implementation program, WHO). This decision was based on three pilot countries where, despite its weak efficacy in phase 3 clinical trial,^2^ the vaccine proved its feasibility to be delivered with a strong safety profile and a high impact in real-life childhood vaccination settings. Moreover, there was no negative impact on health behavior (use of insecticide-treated bednets, childhood vaccinations, and health-seeking behavior for febrile illness). An RTS,S second generation vaccine, called R21 and developed by Oxford Jenner Institute, has shown an improved vaccine efficacy,^3,4^ which meets the WHO goal of 75% of protection in seasonal transmission area following phase 3 trial, and will be soon implemented in the field (WHO recommendation, 25–29 September 2023).

Besides the subunit vaccine development against *Pf*, whole organism vaccine approaches based on attenuated sporozoites (SPZ), or delivery of SPZ under chemoprophylaxis, have shown exciting results against homologous or heterologous controlled human malaria infection (CHMI).^5–8^ These whole-pathogen approaches do not rely on a single protective antigen and thus may circumvent genetic restriction within the human population. Radiation-attenuated SPZ (RAS) have long been known to induce sterile immunity.^9–11^ The delivery of SPZ from a thousand bites of irradiated *Pf-*infected *Anopheles stephensi* mosquitoes in several immunization sessions induced up to 93% sterile protection against CHMI, occurring within 10 weeks of immunization (reviewed in studies by Goh et al. and Hoffman et al.).^12,13^ Immunization via mosquito bites of RAS (IMRAS) has been considered a “gold standard” malaria vaccine. It has led to the discovery of new pre-erythrocytic stage antigen targets^14–16^ and to a better understanding of the immune responses that confer sterile immunity in rodents^17–19^ and in humans.^13,20–22^ Particularly, the IMRAS trial^23^ was designed to achieve 50% vaccine efficacy against CHMI in order to produce a balanced number of protected and non-protected subjects for the purpose of generating a repository of cryopreserved biological samples that could be used to identify an immune signature of protection.

We have previously established the immunological landscape of *Plasmodium*-specific adaptive immune responses of IMRAS trial by performing longitudinal analyses of cellular and serological parameters.^22^ The range of humoral and cellular immune responses was broad after immunization and the frequency of TNFα-secreting SPZ-specific CD4^+^CXCR3^+^ T cells were significantly predictive of protection. Moreover, the machine learning approach using the random forest model identified CD19^+^CD24^hi^CD38^hi^CD69^+^ SPZ-specific transitional B cells, TNFα-secreting circumsporozoite (CS)-specific CD8^+^CXCR3^−^CCR6^−^ T cells, IFNγ-secreting CS-specific CD8^+^CCR6^+^ T cells, and TNFα/IFNγ-secreting CS-specific CD4^+^CXCR3^−^CCR6^−^ T cells as parameters predictive of protection. We wanted to elucidate the immunological pathways involved in RAS immunization by studying the immune response at the transcriptomic level, knowing the involvement of antigen-specific cells in protection. Previous studies analyzed whole blood RNAseq samples at different time-points post-immunization.^24–26^ Although these studies identified Th1 or Th2 polarized T cell responses associated with protection status, further progress was hampered by inter-subject variability and cellular heterogeneity. To reduce nonspecific signals often associated with whole blood transcriptomics and probe SPZ-specific transcriptional response associated with protection, we stimulated peripheral blood mononuclear cells (PBMCs) from pre- and post-immunization and post-CHMI time points after *Pf*SPZ exposure. We then enriched antigen-specific cells by magnetic sorting based on CD69 positive cells, a marker widely expressed on various lymphocyte populations after activation and rapidly upregulated (<2 h).^27,28^ By focusing our transcriptomics measurements only on antigen-specific cells rather than whole blood, we aimed to increase the signal-to-noise ratio of antigen-specific responses over the general inflammatory response and identify pathways triggered by exposure to SPZ between protected (P) and non-protected (NP) individuals over time. Analyzing the multiple timepoints after CHMI was done to identify changes in immune responses due to the parasite’s ability to modulate immunity, especially at the blood stage.^29–31^

## Results

### Sporozoite-specific transcriptional signature of RAS-immunization

Previous studies have reported on the whole blood transcriptional response after immunization with irradiated SPZ (*Pf*SPZ).^24,26^ To potentially increase the resolution of the analysis, we focused our analysis of the transcriptional response on antigen-specific cells by stimulating cells *in vitro* with NF54 *Pf-*SPZ or media control and then magnetically enriching for CD69^+^ cells, an activation marker that can be used as surrogate marker for antigen-specific cells.^27^ After quality control and filtering out lowly expressed genes, 15,923 genes survived for further analysis.

**Figure 1.**
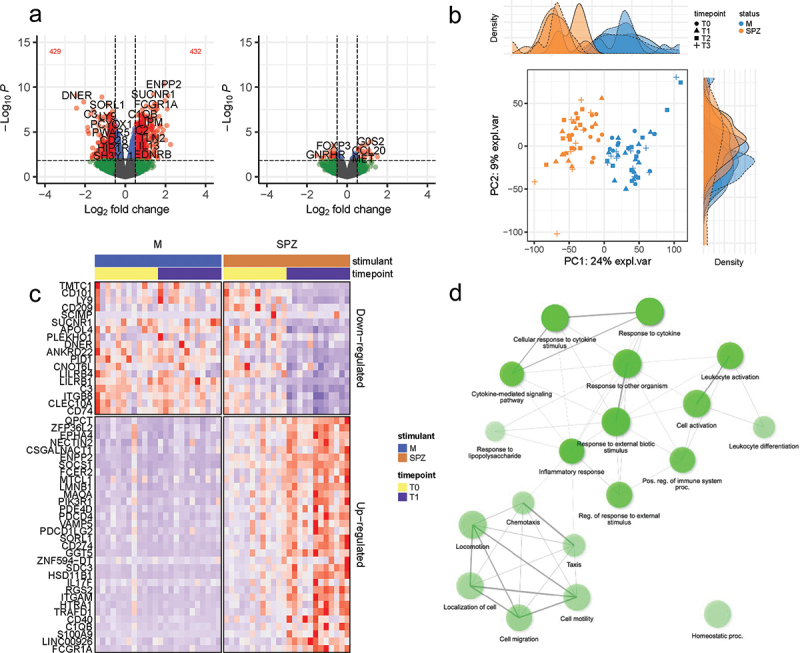
Vaccine-induced changes in CD69^+^ cells after *in-vitro* SPZ-stimulation compared to unstimulated cells. (a) Volcano plot showing differentially expressed genes for pre- (T0) vs post-vaccination (T1) analysis with SPZ-stimulated or unstimulated (M) cells. (b) PCA plot representing each individual at each timepoint (T0, T1, T2, T3) for SPZ-stimulated (SPZ, orange) or unstimulated (M, blue) PBMCs. (c) The top 50 most up- or downregulated genes in response to SPZ-stimulation by *p*-value of univariate significance test (*P* < 5E–7). (D) Network plot showing relationship among enriched GO BP (gene ontology biological processes) terms of SPZ-stimulated samples at T1. The nodes represent BP terms, and the edges indicate shared set of genes between the GO BP terms.

**Figure 2.**
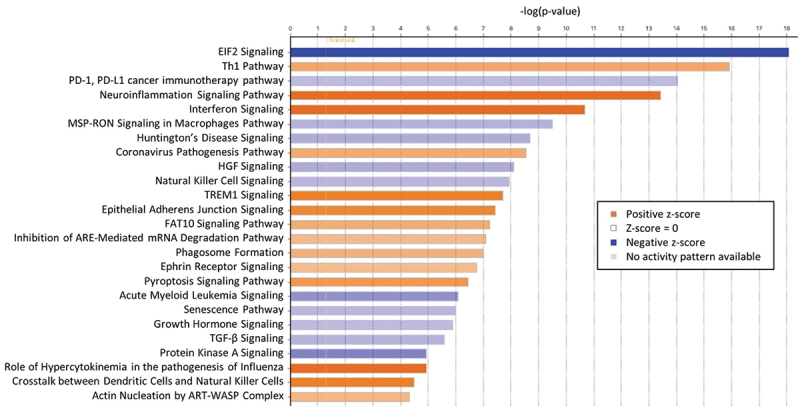
Top 25 canonical pathways enriched for vaccine-induced SPZ-stimulated DEG set (comparing pre- and post-immunization timepoints, *n* = 12). Red and blue colors indicated up- and downregulation, respectively. The intensity of the colors quantified the magnitude of the activation score from IPA analysis.

**Figure 3.**
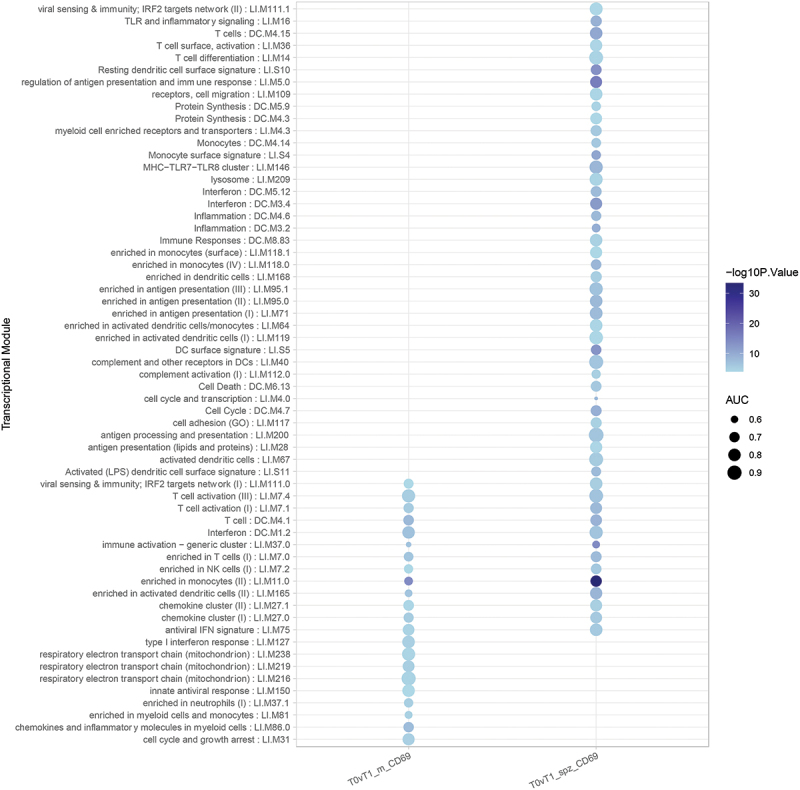
Top enriched gene expression modules in vaccine-induced DEGs set. Many enriched modules were detected only in SPZ-stimulated cells. Area under the curve (AUC) quantified effect-size of the enrichment level from tmod CERNO test.

Comparing transcriptomic profiles of pre-(T0) with post-(T1) immunization timepoints revealed many differentially expressed genes (DEGs), indicating the robust transcriptomic response to IMRAS vaccination. About 432 genes were upregulated, and 429 were downregulated (FDR <0.1, log2 fold-change >0.5 or < −0.5) in the SPZ-stimulated CD69^+^ cells (Figure 1a). We observed that most DEGs induced by immunization were SPZ-specific. The principal component analysis clearly grouped samples regarding the *in vitro* stimulation status (Figure 1b), demonstrating that the systematic differences due to stimulation status were larger than variation due to inter-subject variability. The top up- and downregulating DEGs are shown in Figure 1c for the two timepoints. These genes did not show statistically different expression pattern in the media-stimulated samples at the corresponding timepoints. Pathway enrichment analysis using Ingenuity Pathway Analysis (IPA) software indicated that the top upregulated pathways (-log(p-value)>3 and (z-score)>1.5) were Th1 response, inflammation, and interferon (IFN) signaling (Figure 2). The top downregulated pathways included the Eukaryotic Initiation Factor 2 (EIF2) signaling, the Programmed Cell Death protein 1 and its ligand (PD1/PDL1) pathway, the Macrophage Stimulating Protein and RON Protein Tyrosine kinase (MSP/RON) signaling and Natural Killer (NK) cell signaling (Figure 2). We confirmed these pathways by gene set enrichment analysis (GSEA) using blood transcription modules. About 133 modules were significantly enriched (*p*-value <.05, adjusted with Benjamini–Hochberg correction), including IFN response, activation of monocytes and dendritic cells, antigen presentation, and inflammation (CERNO test, tmod package). The top 30 modules are represented in Figure 3. The network of enriched Gene Ontology Biological Processes (GO BP) terms in response to SPZ-stimulation after vaccination included cell activation, proliferation, cytokine secretion, and inflammation (Figure 1d).

Overall, by analyzing the transcriptional response of antigen-specific cells after immunization, we revealed that several pathways were activated by SPZ-stimulation, including a polarized Th1 response, antigen presentation and IFN response.

### Protection is associated with a larger transcriptomic response to IMRAS vaccination

**Figure 4.**
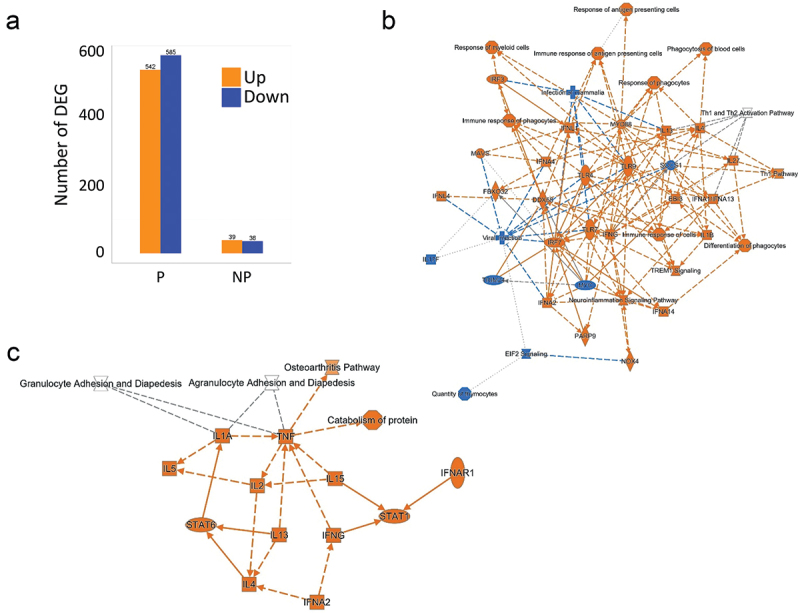
Comparison of vaccine-induced transcriptomic responses in protected (P) and non-protected (NP) individuals. (a) Bar graph representing the magnitude of post-vaccination transcriptomic responses in P and NP subjects (*p* < .05). Colors represent upregulation (orange) and downregulation (blue) (fdr < 0.1, log2 fold change >0.5 or <−0.5, respectively). Numbers on top of graphs represent the exact numbers of DEGs. (b) Canonical pathways (upregulated in orange and downregulated in blue) in the vaccine-induced transcriptional changes for P subjects comparing T1 to T0 using IPA software. (c) Canonical pathways (upregulated in orange and downregulated in blue) in the vaccine-induced transcriptional changes for NP subjects comparing T1 to T0 using IPA software.

The protection status of each individual was determined using CHMI 3 weeks after the final immunization. Comparing the average gene expression profiles between protected (P, *n* = 8) and non-protected (NP, *n* = 4) subjects after immunization (T1), no gene reached statistically significant differential expression difference after multiple testing correction. Nevertheless, intra-individual change between pre- and post-vaccination timepoints in P subjects had a higher number of DEGs (FDR <0.05) than NP subjects (Figure 4a). As sample sizes were imbalanced between the two protection status groups, we sought to assess the effect of sample size differences on the observed difference in the magnitude of the transcriptomic response. We performed differential expression analysis by choosing four random sub-samples from the eight P subjects (50 repetitions, without replacement). The resulting distribution of the magnitude of DEGs confirmed that P subjects had markedly larger transcriptomic responses than non-protected subjects (data not shown).

Therefore, we observed that the size of the transcriptomic response to SPZ-stimulation after immunization is significantly larger in P subjects, as a relatively small number of DEGs were found for NP individuals.

### Vaccine-induced pathway activity patterns/signatures in protected and non-protected individuals

**Figure 5.**
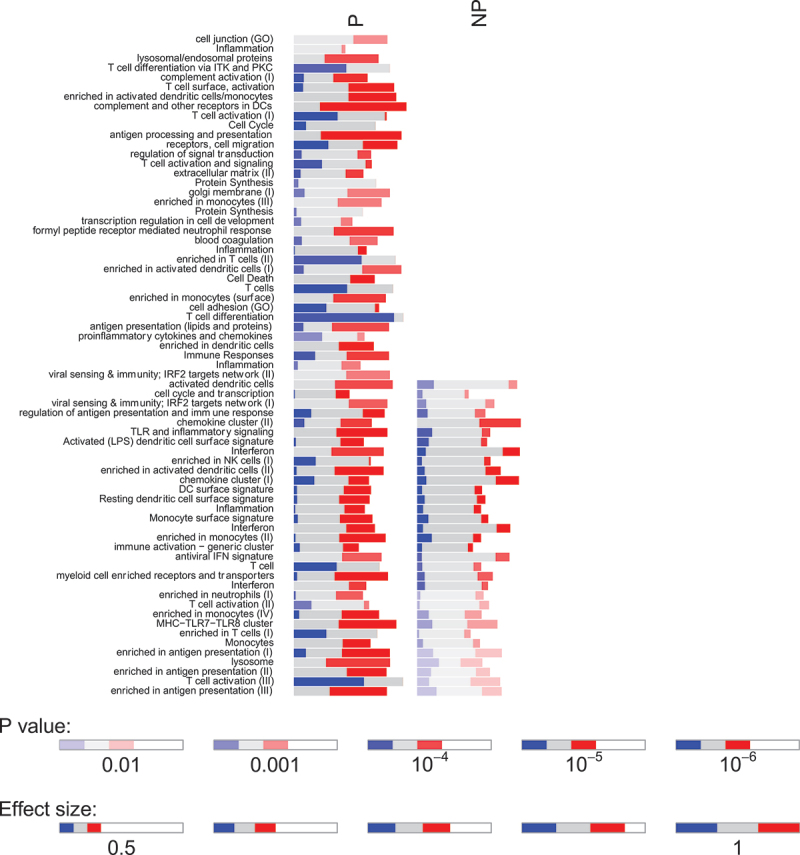
Bar graph representing the gene set enrichment analysis of blood transcription modules in protected (P) and non-protected (NP) individuals in response to IMRAS vaccination (blue: downregulated genes; red: upregulated genes; grey: no significant modification of gene expression). The intensity of the colors and the length of the bars represent association *P* values and effect size (AUC) of enrichment scores.

As NP subjects had few DEGs in response to SPZ-stimulation after immunization, the SPZ-specific transcriptional signature of RAS-immunization described earlier reflected mainly the immune response of protected individuals. IPA analysis of DEGs in P individuals revealed a network of upregulated pathways involving Toll-like receptors and Rig-I like receptors (TLR/RLR) signaling, IFN responses, Th1 polarization, phagocytosis, and antigen presentation, and the downregulation of EIF2 signaling and Il17F (Figure 4b). In NP individuals, only few upregulated pathways were noticed, involving mainly cytokine responses (Figure 4c). GSEA using blood transcription modules confirmed changes in modules involved in antigen processing and presentation, T cell differentiation and activation, as well as cell cycles in P individuals only (Figure 5). We also found several common modules between P and NP individuals although the magnitude of change for NP individuals was smaller.

By assessing the specific transcriptional response to SPZ stimulation *in vitro*, we were able to discern the differential pathway activation patterns of response of P and NP individuals following IMRAS vaccination.

### Modification of the SPZ-specific transcriptional signature of RAS-immunization after CHMI

**Figure 6.**
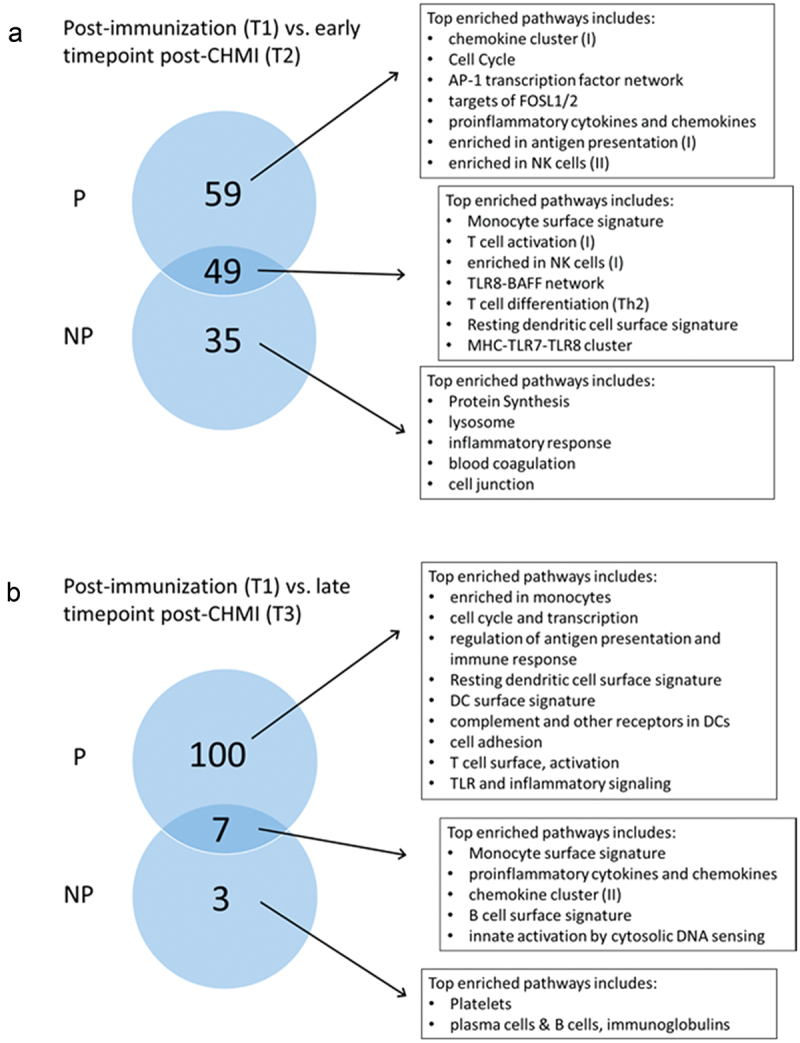
Post-CHMI challenge transcriptomic modifications in protected (P) and non-protected (NP) individuals diverge over time. (a) Circles represented the number of enriched pathways common or specific to P and NP subjects at an earlier timepoint (T2, 5–6 days) post-CHMI challenge. (b) Circles represented the number of enriched pathways common or specific to P and NP subjects at a later timepoint (T3, 3–4 months) post-CHMI challenge.

Finally, we investigated the possibility that CHMI modified the SPZ-specific transcriptional signature of RAS-immunization, due to *Plasmodium’s* ability to modulate the immune response, especially once infection has reached the blood stage. There were only few DEGs that were up- or downregulated when comparing T1 (post-immunization) versus T2 (5–6 days post-CHMI) or T3 (3–4 months post-CHMI). This did not allow an analysis of pathways with IPA software. Thus, we performed GSEA using the tmod package, for P and NP individuals separately. While multiple modules were enriched at T2 compared to pre CHMI challenge (T1) in both P and NP individuals (Figure 6a), the enrichment persisted in P individuals through the later timepoint (T3) but gets attenuated in NP individuals (Figure 6b).

Overall, these time-dependent differences in activation patterns between P and NP individuals suggest a diverging trajectory over time, the difference getting more pronounced over time.

## Discussion

The present study established the SPZ-specific transcriptomic signature after immunization with irradiated parasites and stratified these profiles based on the protective status of the vaccinees. After immunization, SPZ-activated cells displayed broad inflammatory and interferon-associated responses; the adaptive response was polarized toward Th1. EiF2 signaling, PD1-PDL1 and MSP/RON pathways that are involved in the regulation of inflammation and cellular stress responses were downregulated. Stratifying the transcriptomics signature based on protection status revealed a significantly larger number of DEGs in P compared to NP individuals. The transcriptomics profile of NP subjects comprises fewer pathways in response to SPZ stimulation after immunization, involving mainly hypercytokinemia, IL-17 signaling and phagosome formation. Protective and non-protective SPZ-specific response showed diverging trajectory over time, the difference getting more pronounced over time.

Previous work on the whole blood transcriptomic profiles of IMRAS subjects after the first immunization^25^ with a longitudinal follow-up at days 0, 1, 3, 7, 14 and 28 indicated that the early innate responses to RAS immunization influenced the development of the adaptive responses and ultimately the protection from CHMI. A strong acute inflammatory and IFN-associated response to the priming immunization correlated with a lack of protection and was also characterized by a skewing adaptive response toward Th2-polarized T cells. Similar analyses have been done following the five successive immunization sessions, and the transcriptome responses to RAS differed strongly after each immunization.^26^ Notably, as opposed to the first immunization session, the subsequent four immunizations revealed a positive correlation between IFN-associated gene transcription and protection. Nevertheless, the authors described that the biggest factor driving variability in transcriptome and circulating cell phenotype populations was inter-subject variability.^26^ Our approach, using *in vitro* SPZ-stimulation and sorting CD69^+^ activated cells, was designed to study the specific transcriptomic response to *Plasmodium* SPZ induced by RAS immunization. *Ex vivo* transcriptomic signatures reflect the state of the host, whereas antigen-specific stimulation allows the identification of transcriptional events triggered in antigen-specific lymphocytes that could occur *in vivo* after encounter with the pathogen.^32^ Thus, the transcriptome analysis after SPZ-stimulation provides a pathogen-specific readout that is more likely to reflect the disease-specific immune signature that a blood signature directly extracted *ex vivo* from the subject. Furthermore, we enriched antigen-specific cells based on the expression of the activation marker CD69. We previously used CD69^+^ as a marker of antigen-specific cells in an activation-induced marker (AIM) assay.^22,33^ By combining *in vitro* stimulation and CD69^+^ cell sorting, we sought to increase the signal-to-noise ratio, reduce the inter-individual variability and focus on SPZ-specific immune response. Inter-subject variability is an important challenge when analyzing immune response to vaccines. Variability in gene expression of human populations have been observed in many studies (reviewed in^34^) and the causes are not well understood. However, gene polymorphisms, differences in gene methylation due to age, sex, and environmental factors are all considered contributing factors.^35,36^ We observed variability in the magnitude (frequency of CD69^+^ cells in bulk PBMCs, number of DEGs per subject) and the specificity of the response (pathways). Stimulating PBMC *in vitro* with specific antigen (SPZ) and isolating antigen-specific cells prior to processing samples for transcriptomics reduced the individual variability. This may be, in part, due to increasing the specific signal and therefore reducing the nonspecific background (as previously described for flow cytometry analysis).^37,38^ Increasing the signal strength above variability will also reduce the false discovery rate. Another strength of our approach demonstrating technical rigor was the consistency of the results in the longitudinal analysis.

Our study found diverging trajectory over time between P and NP subjects. This is in accordance with early whole-blood *ex vivo* transcriptomic data indicating divergent paths associated with protection, specifically inflammation, T cell polarization, NK cells magnitude, and type-I IFN response.^24^ This divergence was also pronounced early after CHMI, NPand mock individuals sharing similarities of their response compared to P individuals.^39^ Malaria has a complex pathophysiology involving different host tissues, and the dynamic control of inflammation and cell proliferation, mainly monocytes and T cells, appears to be crucial for an effective immune response.^40^ The induction of type-I IFN in response to AT-rich motifs in *Plasmodium* genomes has been well-described.^41^ The exact role of type-I IFN is not clear because it has been associated with protection as well as pathology,^42–45^ but time, source, location and regulation may be the keys that can explain its seemingly conflicting role. After infection, SPZ are rapidly directed to the liver where they infect hepatocytes. The IFN response in the liver is the primary mediator of innate immune control of liver-stage malaria.^46,47^ We observed a strong upregulation of IFN signaling in P individuals, consistent with *ex vivo* transcriptional signatures after the second to fifth RAS-immunization.^26^ Our method reduced inter-individual variability observed in *ex vivo* transcriptional signatures and confirmed the activation of IFN signaling after RAS-immunization in P individuals.

A larger number of pathways were up- or downregulated in P individuals, underlining the magnitude and complexity of the response, as observed for other malaria vaccine candidates. For the RTS,S vaccine, both immunogenicity and vaccine-induced protection was positively associated with plasmablast-associated transcriptional signatures, cell cycle genes, immunoproteasome, and type-I IFN genes. In contrast, NK cell-related genes were negatively correlated with protection and immunogenicity.^48,49^ In a heterologous prime/boost strategy using RTS,S and a viral vector (MVA), TLR-signaling, dendritic cell, and antigen presentation gene expressions correlated with protection, while NK cell genes were again negatively associated with protection.^48^ These vaccine-associated transcriptional signatures involved a complex interplay of pathways that differ notably between vaccine platforms (protein subunit, viral vector, and RAS whole organism approach), but these studies associated protection positively with IFN responses, and negatively with NK cell genes. The IMRAS whole-blood transcriptomic studies revealed that the protection status was associated with different timing, magnitude, and nature of NK cell-related genes early after immunization.^25^ The role of NK cells in malaria immunity, specifically in the antibody-mediated immunity,^50,51^ has been recently spotlighted in Kenya and Uganda,^52,53^ showing a potential link between the maintenance of a subset of natural killer cells (CD56 negative) and the successful control of malaria infection.^53^

When comparing P and NP individuals, we were surprised to find a quantitative difference in the numbers of significant DEGs, suggesting that NP individuals did not notably change their transcriptional profile in response to SPZ-stimulation after RAS-immunization. Nevertheless, all individuals, including P and NP subjects, got a higher relative percentage of CD69^+^ cells in total PBMCs after immunization (T1), and previous studies confirmed that NP individuals have mounted a specific adaptive immune response.^22,23^ While the number of NP individuals in our study is small, it still raises a question about the responsiveness of these individuals to RAS-immunization. Hepatitis B vaccine is known to have 5–10% of non-responders. An *ex vivo* transcriptomic study of the Engerix-B® immunization^54^ revealed DEGs between responders and non-responders before vaccination, related to differences in the levels of granulocytes between the groups. The baseline state of the immune response has also been involved in the responsiveness to influenza vaccine,^55^ and there is now evidence supporting the notion that baseline immune status can predict and potentially affect vaccine responses.^56^ The limited sample size in our study (eight P and four NP subjects) did not allow an adequately powered cross-sectional analysis at baseline.

Our previous study profiling adaptive immune responses revealed that functional cellular responses within the CD8^+^ T compartment and the frequency of antigen-specific transitional B cells were associated with protection. Interestingly, there were several parameters that were distinguishing P from NP individuals at baseline (i.e., before immunization).^22^ Such differences at baseline forecasting vaccine outcome have been reported (reviewed in Tsang et al.).^56^ One implication arising from these reports has been the possibility of modulating the baseline to achieve greater vaccine efficacy. Differences in the baseline responses has also significant bearing when testing a vaccine in different populations, which could explain the differences in vaccine efficacy when testing in U.S. naïve study participants vs field trials where chance for concomitant infections with other pathogens is more likely. A recent review investigated differences in SARS-CoV2 vaccine efficacy and immunogenicity in populations with chronic parasitic infections supporting this hypothesis.^57^

Interpretation of the present study should be done considering the following limitations and strengths. The main limitation is the sample size of the study, owing to the time and resource intensive nature of clinical trial with human challenge. Although our sample size is comparable to similar contemporary studies, it is relatively inadequate to perform a cross-sectional analysis between protection groups at baseline, such as building a machine learning classification and prediction models. Another notable limitation of the study is the fact that the clinical specimens/samples are limited to peripheral blood. Other relevant tissue sources in humans, such as the liver, cannot be accessed for research purposes. On the other hand, our study has several important strengths. First, the *in vitro* SPZ stimulation may more closely mimic the event of pathogen encounter and reveal molecular profiles that are more directly pertinent to the pathology of malaria. Second, focusing on antigen specific cells, instead of the whole blood, reduced individual heterogeneity observed in previous studies done on bulk PBMCs. Third, the longitudinal nature of the study with carefully chosen timepoints enabled controlling inter-individual variabilities that are not directly related to the biological and clinical outcomes of interest.

In summary, our findings demonstrated IMRAS vaccination induced intricate and significant transcriptomic modifications in SPZ-reactive cells present in the peripheral blood. In particular, protective modifications were found to be associated with more robust and larger gene expression changes after completed vaccination. In addition to this quantitative difference between protected and non-protected individuals, we observed a qualitative difference in terms of a diverging pathway activity patterns over time. We identified specific pathways that were significantly activated following immunization and appeared to mediate protection against CHMI. CHMI further drove diverging transcriptomic profiles in protected vs non-protected individuals. Future studies investigating the effect of baseline transcriptomic profiles and background genetic variants on efficacy of IMRAS vaccination would enhance our understanding into genetic factors modulating immune responses associated with protection and susceptibility to parasitic infections.

## Materials and methods

### Study design

Samples for this study, i.e., peripheral blood mononuclear cells (PBMCs), were collected under a clinical protocol (www.clinicaltrials.gov trial ID NCT01994525, accessed on 12 January 2022) from an open-label clinical study for safety and identification of biomarkers of protection in two cohorts of healthy malaria-naïve adults.^23^ Each volunteer got five immunization sessions involving around 200 bites from *Anopheles stephensi* mosquitoes that were infected with PfRAS NF54 (*n* = 21). Leukapheresis samples were available from four timepoints: pre-immune (T0) as a reference sample for each volunteer; post-immune (T1), after the third immunization (pre-CHMI), to establish a signature of immunization; day 5–6 post-CHMI (T2) as an early time point after infectious SPZ inoculation via mosquito bites; and 3–4 months post-CHMI (T3) to determine evidence of editing of the immune response by the CHMI. As leukapheresis was optional during the follow-up of the cohorts, a complete biological follow-up including cryopreserved PBMCs from timepoints T0, T1 and T2 and/or T3 were available for only 12 immunized volunteers with 8 protected (P) and 4 non-protected (NP) individuals after CHMI. The present study was based on these 12 immunized volunteers.

### PBMCs stimulation and CD69^+^ magnetic cell enrichment

Cryopreserved PBMCs from the four timepoints were cultured either in media alone (negative control) or stimulated with sporozoites (SPZ) isolated from salivary glands of *Anopheles stephensi* infected with NF54 *Pf* (15,000 lysed SPZ/per 1 × 10^6^ PBMCs). Cells were cultured for 18 h (37°C, 5% CO_2_) in complete medium (RPMI-1640 (Life Technologies, Waltham, MA, USA) containing 10% human serum (Gemini Bio-Products, West Sacramento, CA, USA)) at a concentration of 1 × 10^7^ cells/mL. CD69^+^ cells were sorted by magnetic enrichment using a positive selection (Miltenyi Biotec, San Diego, CA, USA). We previously demonstrated that enriching antigen-specific cells based on activation markers focuses the analysis on antigen-specific cells.^33,37^ Briefly, cells were incubated with CD69-biotin antibody in Flow buffer during 15 minutes at 4°C, then with anti-biotin microbeads during 20 minutes at 4°C. After washing, cells were enriched on LS columns on an AutoMACS Pro Separator (Miltenyi Biotec, San Diego, CA, USA) directly in Trizol and frozen at −80°C. RNA were extracted using Direct-Zol RNA microprep kit (Zymo Research, CA, USA). RNA quality and concentration were assessed on an Agilent Bioanalyzer (RIN >8).

### CD69^+^ cells RNAseq

RNAseq libraries were prepared using Ovation® SoLo RNA-Seq kit (NuGEN) and quality was assessed by Agilent bioanalyzer. After normalization, the sequencing was done by NCI sequencing facility on NovaSeq 6000 system (Illumina). Demultiplexed fastq were run though a quality control/adapter trimming/read mapping/gene quantification pipeline implemented in Galaxy.^58^ Initial quality control analysis of fastq files was performed using FastQC v0.72.^59^ Read mapping was performed using STAR v2.6.0 and the UCSC hg19 genome annotation.^60^ Counts-per-gene quantitation was performed using FeatureCounts v1.6.2.^61^ The overall pipeline quality control metrics were aggregated and visualized using MultiQC v1.6.^62^ Most samples showed greater than 60% uniquely mapping reads, with a few samples showing slightly higher percentages of reads trimmed too short to be uniquely mapped.

### RNAseq differential expression and gene-set enrichment analysis

The R package edgeR (version 3.42.2) was used for performing differential expression analysis.^63^ After fitting negative binomial generalized linear models, quasi-likelihood F-tests were used to test univariate differential expression of each gene. For comparison between timepoints of the same individuals (e.g., T0 vs T1), paired test was conducted by adding individual IDs in the design matrix. This allowed controlling for inter-individual differences while testing intra-individual changes over time. Significance threshold was set as FDR corrected p-value less than 0.1 and log fold-change greater than 0.5 or less than −0.5.

Two complementary approaches of pathway analysis were conducted. In the first approach, over-representation of pathways in the various DEG sets was tested on Ingenuity pathway analysis (IPA version 1.0 (1.8.0_311)). After uploading the whole genome differential expression analysis statistic on the IPA web interface, a threshold of FDR < 0.1 was set as a cutoff for significance. A result of Fisher exact test was obtained on a curated set of canonical pathways. Pathways with *P* < 1E–3 and |z-score| > 1.5 were considered significant.

In the second approach, gene set enrichment analysis as implemented in the tmod R package (version 0.50.13) was utilized. Unlike the over-representation analysis, instead of using only the DEG set, the whole set of available genes/features ordered according to their significance level were used. In our case the genes were ordered based on their univariate test p-value. Blood transcriptional modules from prior publicly available publications were used on tmod.^64,65^ Statistics were computed using the CERNO test.

Visualizations and plots: the network showing the relationship between enriched GO BP in the T0 vs T1 DEG set was done on shinyGO. Log2 transformed CPM values were used to compute and plot principal components. Modules labeled ‘TBD’ or ‘undermined’ were removed from various plots and tables.

## Data Availability

The data that support the findings of this study are available from the corresponding author.
